# Aspects Supporting and Hindering Type 2 Diabetes Self-Management in Web-Based Educational Portals: Usability Testing Study With Updated Framework in Razavi-Khorasan, Iran

**DOI:** 10.2196/78903

**Published:** 2026-04-01

**Authors:** Javad Jafari, Klas Karlgren, Hossein Karimi Moonaghi, Mohammad Reza Mazaheri Habibi, Saeid Eslami, Italo Masiello, Stefano Bonacina

**Affiliations:** 1 Department of Learning, Informatics, Management and Ethics, Karolinska Institutet, Stockholm, Sweden; 2 Department of Medical Education, School of Medicine, Mashhad University of Medical Sciences, Mashhad, Iran; 3 Health Informatics Centre - Department of Learning, Informatics, Management and Ethics, Karolinska Institutet, Tomtebodavägen 18a, 4th Fl., Stockholm, SE-171 77, Sweden, +46 852483346; 4 Department of Research, Education, Development and Innovation, Södersjukhuset, Stockholm, Sweden; 5 Department of Health and Functioning, Faculty of Health and Social Sciences, Western Norway University of Applied Sciences, Bergen, Norway; 6 Nursing and Midwifery Care Research Center, Mashhad University of Medical Sciences, Mashhad, Iran; 7 Department of Health Information Technology, Varastegan Institute for Medical Sciences, Mashhad, Iran; 8 Pharmaceutical Research Center, Mashhad University of Medical Sciences, Mashhad, Iran; 9 Department of Computer Science and Engineering, Mälardalen University, Västerås, Sweden

**Keywords:** diabetes mellitus, type 2, self-management, patient portals, user-centered design, patient education as topic, web-based learning

## Abstract

**Background:**

The rising prevalence of diabetes necessitates continuous monitoring and treatment, especially for type 2 diabetes. Patient education plays a crucial role in enabling self-management, and web-based educational portals can support patients effectively. While the literature highlights various issues impacting self-management system design, few studies explore the usability aspects that either facilitate or hinder such systems for this patient group.

**Objective:**

This study aimed to investigate usability issues related to a web-based educational portal for patients with diabetes in Razavi-Khorasan Province, Iran. Additionally, it sought to develop a framework of design principles to address critical usability concerns for diabetes self-management.

**Methods:**

The literature on diabetes self-management was analyzed. The analysis focused on design and usability issues affecting self-management. A think-aloud study was carried out with 10 patients using a web-based educational portal designed for patients with diabetes. The portal comprises 9 sections, with patients performing tasks related to each one. Participants’ task completion times were measured, and severity ratings of usability problems were calculated.

**Results:**

Five design principles were proposed that cover (1) understanding and learning about one’s condition, (2) motivation and fostering sustained practices, (3) autonomy and confidence, (4) interaction and collaboration, and (5) privacy and security. The study identified 111 usability problems related to the portal’s 9 sections. Feedback from participants was considered to refine the principles, and participants’ considerations were recorded for any issues that worked against the principles. A total of 16 suggestions for improvement were extrapolated from the data.

**Conclusions:**

This study highlights critical usability and design aspects to consider when developing web-based educational portals to support self-management in type 2 diabetes.

## Introduction

### Background

Diabetes is a chronic disease rapidly increasing in incidence; in 1990, the number of people with diabetes was 200 million but rose to 830 million in 2022, and in low- and middle-income countries, the prevalence has been rising more rapidly than in high-income countries [1], with type 2 diabetes (T2D) accounting for 90% of all diabetes [2]. As there is no cure for diabetes, solutions have focused on preventing complications by educating patients, more recently through online interventions. Web-based patient education interventions may help improve T2D self-management [3]. The increased availability of the internet and smartphone apps targeting patients with diabetes can offer opportunities and improvement [4]. Websites and mobile apps are readily available, cost-effective, and easy for patients to use anywhere and at any time [5]. Furthermore, studies have also shown that internet-delivered diabetes education is easier for patients to access than traditional education [6].

Today, there is a growing number of websites and other forms of online patient education, especially for patients with diabetes [7], and digital diabetes self-management education has been shown to improve patients’ knowledge and clinical outcomes [8]. Many diabetes apps offer a selection of similar functions necessary for diabetes self-management [9]. With the right technical skills, a patient with T2D can take advantage of online patient education and services.

Computer literacy has been defined as “basic skills in using a computer, for example, to save and open a file, use a word processing program, and send and receive e-mail” [10]. Similarly, eHealth literacy is defined as the “ability to seek, find, understand, and appraise health information from electronic sources and apply the knowledge gained to addressing or solving a health problem” [11]. A study recently conducted in Iran concluded that the level of eHealth literacy regarding health technologies is increasing [12]. In addition, according to statistics presented by the Global Economy, approximately 82% of Iran’s 85 million inhabitants were using the internet in 2022 [13], while in 2012, the number was only 53.3% (as identified in the data accessed in 2014 for previous research referenced in a study by Jafari et al [14]). This reveals the rapid increase in the use of the internet in the Iranian population and the need for computer and eHealth literacy for this T2D population to extract the benefits of online patient education and services.

### Designing Tools for Self-Management

Self-care focuses on the prevention of a disease and the maintenance of well-being, while self-management focuses on coping with the disease and controlling the clinical parameters [15]. Research shows that the digital health industry has a promising future [16]. Technology can be used to enhance or regulate people’s emotions, motivation, and well-being [17]. Proactive communication by health care teams can help patients foster autonomy and encourage them to develop better self-management skills [18]. In addition, technology can support patients with chronic conditions by allowing them to increase their control of the disease [19]. There is a need for a supplementary design in developing supportive health care technology, especially for older people with diabetes [20]. However, it is not obvious how all these issues should be implemented in practice when designing web-based resources for the group of patients with T2D.

When self-managing the disease, patients should take responsibility for interacting with health care providers and have the ability to monitor their own condition [15]. Technology designed for diabetes self-management can be helpful in terms of improving blood sugar control, but it is necessary for designers to know how patients use the technology in their daily routines and over an extended period [19].

Quality of life (QoL) is defined by the World Health Organization as “an individual’s perception of their position in life in the context of the culture and value systems in which they live and in relation to their goals, expectations, standards, and concerns” [21]. QoL is fundamental for human-centered design. In human-centered design, trade-offs are general and unavoidable [21]. Design trade-offs are crucial in any design process, particularly as the digital age continues to evolve in the context of T2D self-management. A “trade-off” is a condition that involves losing one aspect or quality of something in return for gaining another aspect or quality [21]. Trade-offs are associated with matching problem solving and problem framing [22]. Designers need to explore QoL [21] and act in the best interests of all stakeholders, including patients. To achieve this, it is necessary to understand the strengths and weaknesses of the technology to be designed [23]. Technology has resulted in progress, but technology adaptation has not improved QoL for patients [22]. An analysis of design trade-offs in developing self-management systems for patients with heart failure concluded that design trade-off analyses can improve understanding of health self-management technologies [24], and analogous analyses are needed for T2D.

### Designing Web-Based Educational Portals

A web-based educational portal is an online tool that allows patients to interact with health care systems and health care providers and follow treatment recommendations for improving their clinical outcomes, while it can also promote preventative measures [25]. In our previous research, a prototype portal was designed and tested for usability in an Iranian context. The development of the web-based educational portal and its preliminary testing has been described in other studies [14,26-28].

Usability is often defined as the “extent to which a product can be used by specified users to achieve specified goals with effectiveness, efficiency, and satisfaction in a specified context of use” [29]. Usability testing is a technique that involves testing products or websites with participants drawn from the target population [30]. Usability testing becomes a crucial part of developing novel health information systems and includes evaluating the degree to which technology fulfills basic usability criteria as the participants perform representative tasks [31]. In addition, usability testing is effective at identifying obstacles and system design issues; therefore, it is highly recommended when developing new health information applications [32]. It is not well known how to design a learning portal to best support patients with T2D in improving their self-care management in an Iranian context—which aspects are important to the target group and how should design trade-offs be managed?

### Study Aim

The aims of this study were to (1) draft a framework of design principles for tools for self-management, (2) explore the usability issues of a web-based educational portal for patients with diabetes, and (3) update the framework of design principles for capturing usability issues important for diabetes self-management.

### Manuscript Organization

In the “Methods” and “Results” sections, we present the development of a theoretical framework with several design principles for exploring usability issues related to diabetes self-management. Then, we present how usability testing was conducted on a web-based educational portal and the findings that have led us to propose a modified framework with an added design principle. Finally, these findings and the framework are discussed and related to other findings in the literature in the “Discussion” section.

## Methods

### Approach to Identifying Design Principles for Diabetes’ Self-Management

We conducted an analysis of our previous work on designing a web-based educational portal to support diabetes self-management [14,26-28] and the literature considered for its development [14,26-28]. The portal was developed for the local context of Razavi-Khorasan, considering regional health behaviors and user preferences [28]. To derive the design principles, we systematically mapped the portal’s functionalities to the 5 layers of influence as outlined in the Ecological Model of Health Behavior [33]: intrapersonal (personalized education and self-monitoring tools to enhance confidence and control), interpersonal (support for family involvement and social engagement), organizational (integration with health care institutions and professional input), community (adaptation to cultural norms and local resources), and public policy (highlighting the need for culturally adapted digital health solutions in low- and middle-income countries). Finally, through a structured, consensus-based approach involving iterative expert panel discussions among the authors and the synthesis of evidence, we consolidated these insights into a set of design principles for web portals that facilitate effective diabetes self-management.

### Study Design and Procedure of the Usability Testing

To describe the usability test, we applied the terminology and the checklist proposed in a study by Martins et al [34]. Following the checklist, we detail the usability assessment moderator, participants, usability evaluation method and usability evaluation technique, tasks, and usability evaluation environment. The checklist filled out for our study is presented in Multimedia Appendix 1. MRMH served as the usability assessment moderator and met the inclusion criteria by having prior experience conducting usability tests. He was external to the portal development team, guaranteeing independence from the product’s design and development process. Finally, no observers were included in the study, and therefore, no observer responsibilities or characteristics were collected.

A sample size of 10 participants was considered appropriate for this usability evaluation based on previous studies [35-37]. They indicate that a relatively small number of participants is sufficient to uncover most usability issues, with Nielsen and Landauer’s model suggesting that 5 participants can identify approximately 80% of usability problems [36]. Extending the sample to 10 participants further increases problem coverage and reliability. Faulkner [37] reported that 10 users can identify approximately 95% of usability issues across typical tasks while maintaining feasibility in formative testing. The inclusion criteria for participation were as follows: having been diagnosed with T2D more than 1 year prior to this study, not having serious diabetes complications, having more than 9 years of formal education, being physically and cognitively able to participate personally in the study, being able to use a computer and internet accessibility, having no experience in using the portal, and, finally, being willing to participate. Participants (ie, patients with T2D) were recruited through convenience sampling from Mashhad Diabetes Clinic. Participant characteristics gathered for the study comprised age, sex, educational level, digital literacy, and the duration of the disease.

We used a single usability testing method using a concurrent think-aloud technique [38], complemented by screen and interaction logging to capture user events (eg, clicks, navigational paths, and errors), without incorporating additional inquiry methods or techniques. In the think-aloud usability test, participants were asked to verbalize their thoughts while completing predefined tasks, allowing insight into their real-time reasoning and difficulties. All sessions were recorded using the computer’s built-in microphone, and participants’ verbalizations were transcribed verbatim in Persian. To document screen activity and user interactions, we used Camtasia Studio (version 8.4; TechSmith Inc.), which enabled synchronized recording of both the event flow and participants’ spoken commentary. Participants completed 9 tasks corresponding to the 9 sections of the web-based educational portal developed by JJ [39], after which they provided severity ratings. Each task targeted a distinct portal section and was performed using the think-aloud technique. Table 1 lists the 9 tasks included in the study. Participants were asked to verbalize their thoughts, feelings, opinions, and experiences while completing the tasks [40]. In individual sessions, participants were first introduced to how the think-aloud method would be used and then received a brief introduction about the portal before the usability test by MRMH. A scenario (Instructions to participants, Multimedia Appendix 2) was prepared based on the results of our previous research [26,27] and was pilot tested with 2 patients prior to the real usability testing. Participants used a laptop running Windows 7, and their activities during the usability tests were video recorded and audio recorded (as abovementioned). During the sessions, the usability assessment moderator (MRMH) sat beside participants to encourage continuous verbalization and to provide assistance if needed—for example, in cases where instructions were unclear, medical terminology was difficult to interpret, or a technical glitch occurred.

**Table 1.** The 9 tasks and their explanations, including the collected data or material.

Task name	Task explanation
Fill in patient information	The user is asked to type or select from a drop-down list. The data are entered into the system using the keyboard and mouse. They include name and surname, gender, age, education level, occupation, address, weight, height, type of diabetes, age of onset of diabetes, and associated specific disease.
Register laboratory results	The user is asked to type laboratory result values and read medical documentation. FBS^a^, 2hpp^b^, 5PM^c^, HbA1c^d^, liver function tests (SGOT^e^, SGPT^f^, ALP^g^), lipids profile (cholesterol and triglyceride), kidney function tests, urine sugar, BUN^h^, creatinine, and blood pressure.
Attending educational courses	The user is asked to attend a course aiming to help them improve their diabetes self-management. Eight courses are available, and they were asked to attend one of these courses. Each course contains a short quiz, which is answered before beginning the course.
Read educational material	The user is asked to read educational material about various aspects of diabetes and its effects on organs and the relationship between stress, physical activity, drugs, and disease progression.
Use the “help-me” steps	The user is asked to follow the “help-me” flow or to follow sequential steps to access specific information.
Check health improvement	The user is asked to access data about their current situation based on their medical condition and the laboratory results recorded in the medical record.
Play educational movies	The user is asked to find and watch one short movie about diabetes for at least 1 minute, regarding diabetes complications, diets tailored for patients with diabetes, or other related subjects. Movies are about 3 to 4 minutes long.
Calculate health indices	The user is asked to use a food calorie counter and a calculator to check the BMI and the glycemic index of different types of food. The task was composed of one calculation based on the user’s interest.
Chat with physicians and nurses	The user is asked to use the chat to communicate with physicians or nurses.

a FBS: fasting blood sugar.

b 2HPP: sugar level 2 hours after breakfast.

c 5PM: 5-afternoon blood sugar.

d HbA_1C_: hemoglobin_ A1c_.

e SGOT: serum glutamic oxaloacetic transaminase.

f SGPT: Serum glutamic pyruvic transaminase.

g ALP: alkaline phosphatase.

h BUN: blood urea nitrogen.

As for the usability evaluation environment, the study was conducted under controlled conditions in a room located in the Medical Informatics Department of the Faculty of Medicine of Mashhad University of Medical Sciences, Mashhad, Iran. This setting was selected because it provided a quiet, interruption-free environment that ensured consistent testing conditions across all participants.

### Data Analysis

The analysis phase integrated quantitative and qualitative components: quantitatively, we computed time on task (summarized with descriptive statistics); qualitatively, think-aloud verbalizations and moderator notes were subjected to qualitative content analysis [41], with iterative code development. A particular focus was on the observations of the participants’ behaviors and their comments related to self-management and the themes of the framework discussed above. Attention was paid to how the web-based educational portal supported or hindered patients’ self-management activities (eg, due to lack of functionality). The time the participants spent on each task and the issues encountered were collected by Camtasia and analyzed. Descriptive statistics, including means and SDs, were calculated for the time taken by participants to complete the tasks. The usability tests were transcribed, and the resulting data were analyzed through qualitative content analysis [41]. Following the completion of the think-aloud sessions, the severity of identified usability problems was assessed by 3 reviewers: 2 members of the team involved in developing the web-based educational portal (AJ and NF; see Acknowledgments) and the first author (JJ). Severity ratings were assigned using Nielsen’s standardized 0‐ to 4-point scale [42], where 0 indicates “not a usability problem,” 1 indicates “cosmetic problem only,” 2 indicates “minor usability problem,” 3 indicates “major usability problem,” and 4 represents a “usability catastrophe” that must be resolved prior to release. Ratings were based on 3 factors: frequency (ie, how often the problem occurred), impact (ie, the extent to which the problem affected task completion), and persistence (ie, whether recovery was easy or difficult). The 3 reviewers (coders) worked independently, and coding agreement on severity scores was reached by consensus.

### Ethical Considerations

All participants received oral and written information about the study and signed a consent form. They were informed about how the gathered data would be used, that their identities would remain anonymous, and that they had the right to withdraw from the study without having to explain why at any time, if they wished to do so. Participants did not receive any compensation. The Ethics Committee of Mashhad University of Medical Sciences approved the study (IR.MUMS.REC. 1395.108; Multimedia Appendix 3).

### Generative Artificial Intelligence Disclosure

Microsoft Copilot has been used to shorten the abstract to the allowed word limit. After using this tool, the authors reviewed and edited the content as needed and take full responsibility for the content of the publication.

## Results

### Overview

The results are presented in 3 sections. First, the first iteration of the framework (“Design Principles for Diabetes’ Self-Management”) is outlined, and then, quantitative and qualitative results from the usability tests are explained, and finally, a revised framework is detailed pertaining to design and usability issues in the context of patient self-management of T2D.

### Design Principles for Diabetes’ Self-Management

#### Initial Version of the Framework

By the approach explained in the *Methods* section, we proposed 4 design principles for diabetes self-management. The first principle concerns supporting patients’ *understanding and learning about the complexities of their condition*. This principle is about creating awareness and supporting patients’ learning regarding how to manage their diabetes, including daily measurement routines, diet planning, exercise, medication, illness, alcohol consumption, and stress. When patients do not understand their condition and the importance of managing it, adequate self-management is at risk and is likely to be discontinued. Patients require support on different levels, ranging from performing procedures, such as measurements, to reflecting on how to plan their lives on an overall level to maintain adequate self-management. Patients with diabetes need opportunities to learn specific skills, and environmental and policy contexts influence the psychological factors involved in diabetes management [33]. Opportunities to learn about measuring blood sugar, planning diets, exercising, and addressing challenges specific to diabetes are helpful for supporting self-management and managing diabetes [33].

The second design principle relates to supporting *motivation and fostering sustained practices*. This principle highlights the importance of supporting patients in keeping up their self-management over time and introducing measures to help them avoid skipping self-care practices or giving up entirely. Encouraging, reminding, supporting, and “nudging” can provide the motivation that patients may need to continue their self-management and not become discouraged or distracted. Plans, calendars, schedules, timely reminders, praise, and even rewards could be elements that contribute to keeping patients motivated and supporting them in modifying their everyday practices. Providing updated information, especially information tailored to the individual patient, and giving feedback may maintain patient interest and thereby help them continue to pursue self-management plans over time [33]. Technologies that suggest care activities or adjustments to current treatments based on information on improved care and using these technologies seem to be positive for patients [19]. Mobile apps help patients to self-manage the disease and motivate patients to improve glucose control [4]. Researchers based on numerous studies “recommend designing for continuity in time, space, and the aesthetic dimension and recommend focusing on understandability and learning” [20].

The third design principle concerns the patient’s feelings of *autonomy and confidence*. Patients do not only need knowledge and reminders but also need to feel confident and secure when it comes to being able to manage their condition on their own. They need to feel that they can manage their T2D without risking their health and know that they can find and obtain help when needed. It is therefore essential to help patients develop a sense of being in control with clear instructions and, at the same time, help them understand that they have the ability to independently plan their self-management without feeling that their role is just to follow instructions. In previous research, patients with heart conditions have expressed the need to acquire knowledge while also building up their confidence to be effectively involved in decision-making concerning their treatment [43]. Technology, if well designed, can be used to contribute to making patients feel more confident about their disease management, for example, by monitoring patient vitals, giving advice on lifestyle changes, and providing relevant patient education materials [19].

**Figure 1.**
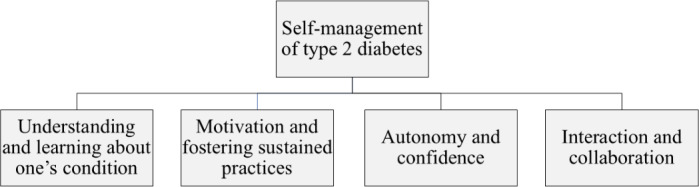
The 4 design principles of diabetes self-management. Results from data collected from the usability testing of the portal.

The fourth design principle concerns *interaction and collaboration* with other patients and health care personnel. Supporting contact and communication between patients and health care providers is a key part of supporting patients’ self-management [33]. Technology can be a vehicle for sharing information between patients and their caregivers and can provide support. However, technology can also create opportunities for patients to share care activities with informal carers and can contribute to engaging in self-management activities and learning from others with the same chronic condition and enhance collaboration with the care team [19]. Being able to connect with others in a similar situation may be an urgent requirement. Sharing experiences and taking part in the experiences of others are strong motivators for using social media and are also powerful forces that can be of interest when designing tools for self-management. Figure 1 shows the 4 design principles. These principles draw attention to issues that are relevant for supporting—or hindering—T2D patients’ self-management. These principles have served as a background and lens for further developing our framework.

#### Description of the Participants’ Characteristics

Ten patients from a diabetes outpatient clinic participated in the study. The study participants were 5 males and 5 females with T2D, and the average age was 46.5 (SD 6.4) years. Participants had a mean disease duration of 7.9 (SD 4.5) years from the time of diagnosis. Seven of the 10 participants had received an undergraduate level education (9‐11 y of education), and 3 had completed postgraduate education (14‐16 y of education).

#### Time Spent on Different Tasks and Severity Ratings

The 10 participants completed the usability tests in a mean time of 2068 seconds (34 min and 28 s; Table 2; SD 212.8). The first task, “Fill in patient information,” took 513 (SD 131.1) seconds (8 min and 33 s) on average to complete, while it took 442 (SD 33.0) seconds (7 min and 22 s) to complete the second task, “Register laboratory results.” The first 2 tasks took longer to complete than the other tasks. Two other tasks on which the participants spent more time were “Calculate health indexes” (task 8) and “Use the ‘Help-me’ steps” (task 5). Table 2 presents the times that each participant spent completing the tasks and the average time and the SD for each task. In addition, it presents “range” and “range width” for each task completion. As mentioned, participants spent more time on task 1 (mean 513, SD 131.1 s; 25%) and task 2 (mean 442, SD 33 s; 21%) and the least time on task 6 (mean 91, SD 19.7 s; 4%), “Check health improvement,” and task 4, “Read educational material” (mean 82, SD 81.5 s; 4%).

**Table 2.** Duration of task completion for each user.

	Task 1—Fill in patient information		Task 2—Register laboratory results		Task 3—Attending educational courses		Task 4—Read educational material		Task 5—Use the “Help-me” steps		Task 6—Check health improvement		Task 7—Play educational movies		Task 8—Calculate health indexes		Task 9—Chat with physicians and nurses		Test duration	
	Value (s)	%	Value (s)	%	Value (s)	%	Value (s)	%	Value (s)	%	Value (s)	%	Value (s)	%	Value (s)	%	Value (s)	%	Value (s)	%
Participant A	610	28.6	460	21.6	270	12.7	30	1.4	200	9.4	90	4.2	120	5.6	210	9.9	140	6.6	2130	100
Participant B	330	15.5	440	20.7	210	9.9	30	1.4	220	10.3	100	4.7	150	7.0	470	22.1	180	8.5	2130	100
Participant C	510	25.6	450	22.6	240	12.1	50	2.5	95	4.8	90	4.5	105	5.3	300	15.1	150	7.5	1990	100
Participant D	390	20.4	480	25.1	200	10.5	60	3.1	240	12.6	60	3.1	90	4.7	300	15.7	90	4.7	1910	100
Participant E	570	22.5	470	18.5	310	12.2	275	10.8	280	11.0	60	2.4	80	3.2	390	15.4	100	3.9	2535	100
Participant F	630	36.5	405	23.5	90	5.2	45	2.6	60	3.5	85	4.9	60	3.5	220	12.8	130	7.5	1725	100
Participant G	490	25.5	420	21.9	230	12.0	50	2.6	250	13.0	60	3.1	80	4.2	190	9.9	150	7.8	1920	100
Participant H	760	35.8	480	22.6	230	10.8	80	3.8	160	7.5	70	3.3	110	5.2	140	6.6	90	4.2	2120	100
Participant I	400	19.5	380	18.5	270	13.1	90	4.4	120	5.8	85	4.1	110	5.4	480	23.4	120	5.8	2055	100
Participant J	440	20.4	435	20.1	195	9.0	200	9.3	380	17.6	120	5.6	110	5.1	180	8.3	100	4.6	2160	100
Time
Average	513	25.0	442	21.0	224.5	11.0	91	4.0	200.5	10.0	82	4.0	101.5	5.0	288	14.0	125	6.0	2068	—
SD	131.1	—^a^	33.0	—	59.2	—	81.5	—	95.4	—	19.7	—	25.2	—	122.3	—	30.3	—	—212.8	—
Range	330‐760	—	380‐480	—	90‐310	—	30‐275	—	60‐380	—	60‐120	—	60‐150	—	140‐480	—	90‐180	—	1725 - 2535	—
Range width	430	—	100	—	220	—	245	—	320	—	60	—	90	—	340	—	90	—	810	—

a Not applicable.

**Figure 2.**
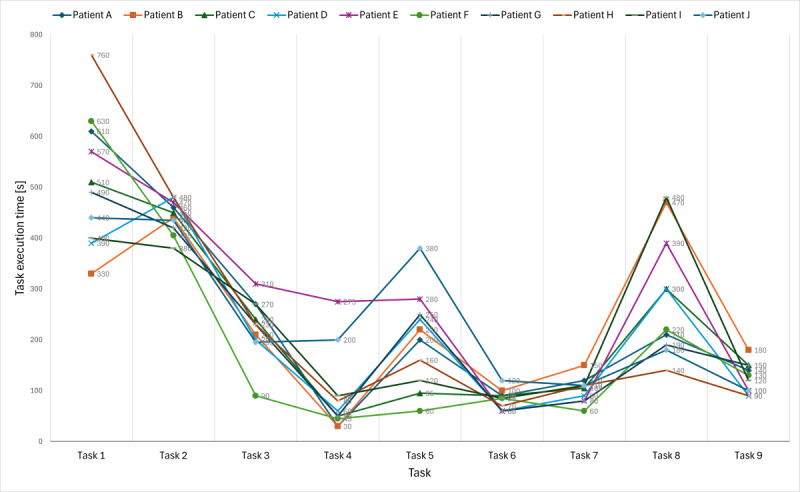
Comparison of the duration of task completion for each user.

Figure 2 presents a comparison of the duration of task completion for each user.

Five tasks were expected to be particularly informative and relevant to the self-management of diabetes:

- Task 3: “Attending educational courses”; take one pretest and look at the courses.
- Task 4: “Read educational material” about diabetes and body organs.
- Task 5: ”Use the ‘Help-Me’ steps” module and follow the steps.
- Task 7: “Play educational movies” and watch one educational video.
- Task 8: “Calculate health indexes,” namely, BMI and daily calorie intake.

Analysis of tasks 3, 4, 5, 7, and 8 showed that participants reflected during task completion (Table 2 and Figure 2) and provided both feedback and opinions. For example, participants pointed out their interest in finding trustworthy and helpful information, educational materials, and short educational videos about diabetes. In addition, they expressed an interest in finding information that could help them solve diabetes-related complications. Participants pointed out the importance of knowing about healthy foods in all food groups and calculating their daily calorie intakes to create healthy-eating plans relevant to their daily activities. These topics are important for diabetes self-management.

Table 3 presents the number of usability problems participants encountered and the problems that were detected in this study according to severity ratings, which range from 0 to 4, determined by the researchers involved. On the basis of the think-aloud method and the qualitative content analysis, 111 problems were detected that show strengths and limitations derived from the usability testing, and some were repeated by participants. Most usability issues (n=46) were classified as minor with a severity rating of 2 (Table 3), whereas 10 issues were rated as severity 4, indicating catastrophic problems. In addition, the participants encountered 14 issues that were not considered usability problems.

**Table 3.** Usability problems were identified by users in terms of tasks and their severity ratings.

Tasks	Severity, n (%)					Total usability problems per task, n (%)
	0	1	2	3	4	
Fill in patient information	8 (21)	10 (26)	10 (26)	6 (16)	4 (11)	38 (34)
Register laboratory results	4 (15)	4 (15)	14 (52)	2 (7)	3 (11)	27 (24)
Attending educational courses	1 (20)	1 (20)	1 (20)	2 (40)	0	5 (4)
Read educational material	0	3 (100)	0	0	0	3 (3)
Use the “Help-me” steps	0	0	3 (75)	1 (25)	0	4 (4)
Check health improvement	0	0	0	0	2 (100)	2 (2)
Play educational movies	0	0	3 (75)	1 (25)	0	4 (4)
Calculate health indexes	0	1 (8)	7 (59)	3 (25)	1 (8)	12 (11)
Chat with physicians and nurses	1 (6)	5 (31)	8 (50)	2 (13)	0	16 (14)
Total usability problems	14 (13)	24 (22)	46 (41)	17 (15)	10 (9)	111 (100)

#### Participants’ Suggestions for Improving the Portal

In total, 16 suggestions on how to improve the web-based educational portal were noted. In Table 4, the suggestions are sorted in ascending order based on their frequencies.

**Table 4.** Suggestions for the improvement of the portal.

User suggestions	Frequencies, n (%)
Need to have a user guide or guideline for the portal	9 (19.1)
Use appropriate messages to interact with the user	8 (17.0)
Embed the list of physicians and nurses in the online chat	5 (10.6)
Use of larger font and readable colors	5 (10.6)
Mark clearly mandatory fields	3 (6.4)
Add more educational content	3 (6.4)
Offer a diet plan	3 (6.4)
Change the titles of the calculator option	2 (4.3)
Do not use medical terms or jargon	2 (4.3)
Classify the content according to patients’ literacy level	1 (2.1)
List specific diseases that a user can select from a list	1 (2.1)
Complete the educational courses	1 (2.1)
Use more pictures and graphs in the educational materials	1 (2.1)
Use simple options to calculate BMI	1 (2.1)
Use more simple options to calculate calories	1 (2.1)
Add an option for booking time to see their physician	1 (2.1)

### A Framework of Design Principles

A large number of observations were made regarding the specific complexities and challenges of using the web-based educational portal, which could affect the self-management of patients with T2D. One type of observation concerned difficulties in terms of finding information and following instructions due to poor usability. Another issue related to poor computer literacy adversely affected the possibility of using the portal for self-management due to difficulties with rather basic interactions with buttons, navigation, and finding information in the portal. In some cases, patients needed more time to complete the tasks than expected due to their lack of familiarity with the portal. Various bugs and errors in links, buttons, and forms were also noted, impacting the portal’s usability. The first section below presents findings relating to the framework of design principles and is followed by data regarding the time spent on the different tasks (explained in Table 1), severity ratings, and suggestions from participants. In total, 111 codes were derived from analyzing the data that pertained to how the design of the portal supported or hindered self-management behaviors. The results are presented together with the framework of 5 design principles (Figure 1).

Regarding the first design principle about supporting patients in understanding and learning about the complexity of the condition, participants emphasized the importance of finding information and pointed out that interaction within the portal could help them learn more about self-management skills. For example, they expressed that they wanted to learn how to calculate their calorie intake and diets and how to use the BMI calculator. Participants appreciated the educational content as being helpful for dealing with self-management problems but also asked for more information about topics that they would have liked to learn more about, such as diet plans and the role of stress:

*Here* [points to the educational section of the portal], *educational content is related to diabetes and body organs that users can [use to] solve their problems.*

*I think it is great; this information helps us know what we have to do because a diabetic patient has a lot of problems.*

*There is nothing written about stress and how it affects the body—I know that stress has a big effect on me.*

While participants appreciated learning about T2D, they pointed out that they had difficulties understanding certain medical terminology, language, and abbreviations on the portal. For 8 of the participants, the term “urine sugar” (glycosuria) was unclear, and for 4 of them, “urea in the blood” (blood urea nitrogen) presented a comprehension problem. All the laboratory items were in English, and it was difficult for participants to enter data about these items. In addition, the use of the abbreviation “BP” for blood pressure was not obvious to users during data entry.

Moreover, participants had difficulties navigating the web-based educational portal to make use of the educational material and in knowing what to do with the information. Six participants had difficulties finding the educational courses and pretest questions on the computer screen. In addition, it was not clear what to do after answering the pretest questions, or, in some cases, the proposed answers were not fully understood. One participant also had an issue with the font size of the text (too small), and 1 patient experienced difficulties accessing the educational course. One participant complained about slow scrolling when studying the educational material. Another participant also complained about the font size during this task. These usability issues risked working against the design principle of supporting understanding and learning. Suggestions from participants included avoiding medical “jargon” and classifying content according to patients’ literacy levels.

The second design principle emphasizes supporting motivation and fostering sustained practices. Regarding reminders about how to go about carrying out measurements in their daily lives—such as calculating calories and creating diet plans that are easy to implement—participants expressed that functionalities of the web-based educational portal could help them modify their everyday practices. Features such as reminders, calendars, schedules, rewards, and praise were considered functions that could support participants’ motivation by “nudging” them into trying new behaviors and maintaining practices over time. Several participants brought up how educational materials about diabetes could support them in maintaining their interest in the portal. They also expressed how accurate and up-to-date facts about their condition would make them feel that the portal was a place they could return to for resources and advice and something that could assist them in not giving up on self-management. Participants appreciated the idea of finding information about treatments, and knowing that they would find such information on the portal would encourage them to return to consulting the portal. A function that was appreciated was the opportunity to check one’s health improvement based on laboratory results, but as the results came from a 3-month data collection period, the participants were not able to see the curve charts on the health progress screen and were therefore not able to test it fully:

*If that was the case, for example, I would input the food and the site would tell me how much to eat based on my height and weight.*

Several comments and observations highlighted the risks of working against this design principle. As mentioned earlier, participants expressed that they wanted to learn how to calculate their calorie intakes and plan their diets, but they also pointed out that they were hesitant to insert information about their treatment or repeatedly import their weight and height into the BMI calculator, as it was experienced as burdensome. Several interactions, especially relating to inserting data, were in fact experienced as troublesome and referred to as “frustrating.” Four participants had difficulties logging in, and 6 participants found it complicated to register, get login codes and passwords, or enter registration information. For all participants, it was unclear what to write in the “patient-specific illness” box in the enrollment portion of the portal, and 4 participants had difficulty entering data into the data entry boxes. Five participants complained about the font size, and 4 of them did not know the date of the onset of their illness, making interaction difficult. All such experiences were considered annoyances that would lessen their enthusiasm in terms of using the web-based educational portal over time. The participants also experienced difficulties entering laboratory test results, which is a recurring activity that should feel easy to carry out. Eight participants experienced problems entering the value of their “sugar level 2 hours after breakfast (2hpp).” Nine participants had difficulties entering the results of the liver tests such as serum glutamic oxaloacetic transaminase, serum glutamic pyruvic transaminase, and alkaline phosphatase, their five in the afternoon blood sugar (5 PM), and the protein in their urine. Six participants had problems entering the value for serum creatinine, and 3 participants had issues entering their cholesterol and triglyceride levels. A default date was included at the end of the laboratory tests, and it made it difficult for participants to enter the actual laboratory date on the portal.

The third design principle concerned autonomy and confidence. As soon as the participants had understood how to use the web-based educational portal, they explained how it made them feel more secure and willing to continue using it. Participants commented on having mastered carrying out certain steps that they were unable to do before. Being able to use the web-based educational portal without the help of others was reported as satisfying:

*I know what I have to do in this step. I did this step and can do the next step; others cannot do the tasks the way I’m doing them.*

*This is the one I can do, and I think it is not difficult for me. It is not necessary to ask someone and get help.*

The step-by-step instructions were appreciated and seen as helpful by the participants in learning how to make use of the portal’s features. In contrast, participants were disappointed when they were not able to follow the sequential steps of the Help-Me screen to get the necessary information, and they called for an additional step that would more clearly show them how to complete the task. More user guides were suggested as being potentially helpful for building up competence in fully making use of the intentions of the portal.

The fourth design principle highlights the importance of interaction and collaboration with others. An online chat function is provided by the portal, which enables communication between patients and physicians or nurses. Having the possibility to contact health care professionals was viewed as important, and one of the participants commented as follows:

*I can find the information that I need on this portal. It is great to have contact with nurses and doctors through this portal.*

*How good it is to be able to contact a nurse online! Well, I do not see anything like a doctor here. It is easier to have the doctor’s name. It would also be interesting to talk to people who have the same problem as us.*

Participants suggested extensions regarding how they could get in touch with health care professionals, for example, by adding the ability to book a time to meet their physician through the web-based educational portal. Some participants had difficulties finding the names of the physicians or nurses on the lists that were available online, and one suggestion was to embed such a list in the chat function. One concern that was voiced was that the text-based interaction between nurses and physicians should occur on an appropriate, understandable level. Moreover, the distinction between scheduled online face-to-face meetings and offline, or asynchronous, communication with physicians or nurses was unfamiliar to the participants.

In addition, several observations were made that did not match the framework’s 4 principles. A common thread was these observations related to the participants’ concerns about their integrity and trusting the system with health data. Participants expressed that they preferred not to share certain information and were uncomfortable being prompted to insert such information. Therefore, the addition of a fifth design principle to the framework relating to security and privacy issues was suggested. Some participants were hesitant about inserting data about their treatments, weight, and height, and there was another aspect to this hesitance. Two participants were unwilling to enter personal information and expressed concerns about security and information privacy and wondered who would be able to see and access the personal data uploaded into the system. The participants referred to the fact that their health information would be in a system that is online as the reason behind their worries. Such concerns, therefore, counter the goal of using the portal. Regardless of whether the users are able to make use of the intended functionalities provided by the system, a lack of confidence in the system risks undermining the overall use of the portal:

*Is it necessary to enter all this information to register? It would be better if the icons had a star to show which ones are necessary. Because I don’t like that, and I don’t register myself on some sites that take all my personal information.*

**Figure 3.**
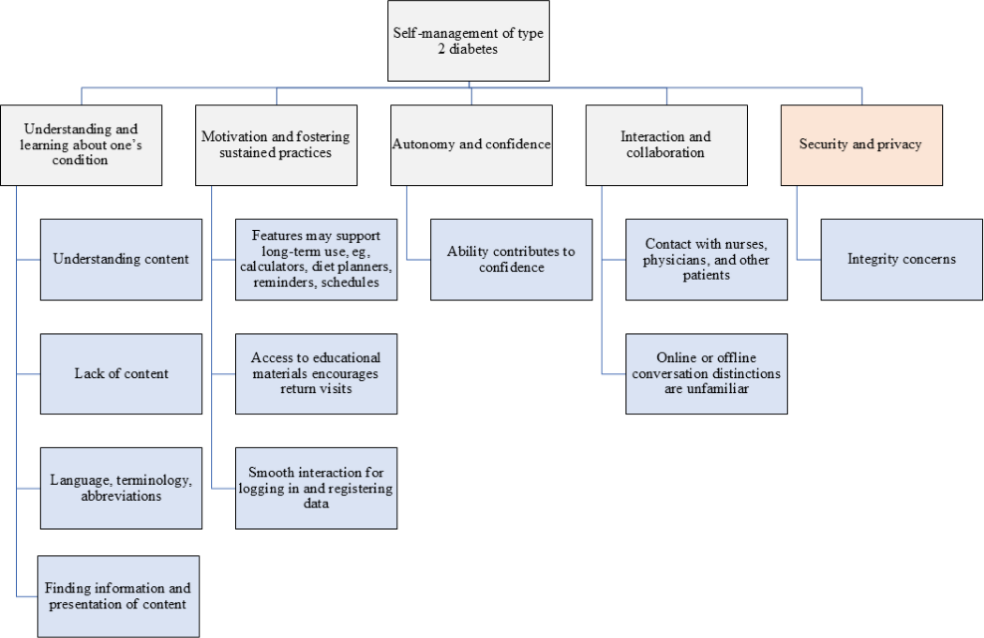
The updated framework.

Figure 3 presents the updated framework, which includes 5 principles.

## Discussion

### Principal Findings

The principal results are structured as follows: a brief summary of the usability testing is provided first, followed by a detailed discussion of the proposed framework.

The usability testing revealed issues affecting user performance and understanding across the evaluated 9 tasks. Participants successfully completed most tasks, but tasks 1 “Fill in patient information” and 2 “Register laboratory results” required more time and caused confusion about terminology (also because laboratory variables were expressed in English). Think-aloud responses showed that participants reflected on unclear instructions and insufficient system feedback for tasks 3, 4, 5, 7, and 8 (Table 2 and Figure 2). Using Nielsen’s severity rating scale, we identified 46 minor usability problems with a severity rating of 2 and 10 major problems with a severity rating of 4 (Table 3). Minor problems were primarily related to tasks 2, 1, and 9, whereas major problems were related to tasks 1 and 2, related to insufficient support for self-management actions and unclear terminology. Suggestions on how to improve the web-based educational portal were noted (Table 4) and were related to providing a user guide and adopting a suitable terminology for the user. Positive feedback highlighted the usefulness of certain information modules (see section “A Framework of Design Principles”). Overall, these findings guided the refinement of the framework by contributing an additional design principle and providing further detail to existing principles.

This study has proposed a framework of design principles and investigated design and usability aspects of a portal aiming to support the self-management of patients with T2D. The design principles emphasize aspects that are important for the self-management and can guide design work. Design principles have been described as providing a bridge between theory and practice while simultaneously playing several possible roles [44]. They can, for example, be a means of explaining, communicating, and discussing criteria for making selections; suggestions for developing existing practices; a framework for reporting findings; or ideas regarding tool functionalities. In this study, the design principles have fulfilled several of these roles and have provided a way of focusing on aspects of the web-based educational portal that contribute to the overall goals of self-management, as well as to the study and evaluation of the proposed solutions.

These aspects are expected to be crucial to consider when designing tools for T2D self-management, and many have been previously discussed in the literature [4,17-20,33]. Here, the principles have helped us concretize these aspects to empower a specific patient group. The importance of the ideas underlying the principles was in part confirmed by the many observations, but many findings have also added to the understanding of the patient group and its needs. The findings contribute to refining the principles, and negative observations and input from participants have also highlighted the risks and drawbacks of design proposals. Regarding the first design principle, emphasizing the patients’ understanding and learning about their condition, we found overwhelming support for patients’ interest in learning more about their condition and improving their self-management skills. The educational materials that were provided were appreciated and considered helpful for dealing with self-management problems, but many more topics were called for. Several usability issues also risked countering this design principle, including poorly designed interaction, navigation, presentation, and the use of language and terminology.

The second principle highlights the importance of supporting motivation and fostering sustained practices. Features of the web-based educational portal, such as reminders, calendars, and calculators, which could support, remind, encourage, maintain, and help participants plan their everyday practices, were considered motivating and helpful. However, participants also pointed out that knowing that the portal offers up-to-date educational materials was something that encouraged them to return to it. Poorly designed interaction, especially when registering data, was considered irritating and was identified as a risk when it comes to the sustained use of the portal.

The third principle focuses on patients’ autonomy and confidence. Being able to carry out tasks on the portal without help from others was clearly pleasing for participants and contributed to a feeling of confidence. However, there is also a need to educate and guide the patients in how to use the system properly and efficiently. This could be done at the health care centers, where a number of one-to-one sessions with a nurse could be scheduled to direct the patients toward autonomy of use. The usability study shed light on an aspect that we had not previously focused on, namely, the confidence users have in the portal. Confidence in the system and how personal data are handled was a crucial issue for some users, and uncertainty regarding data security was something that risked undermining patients’ use of the portal.

The fourth design principle highlights the importance of interaction and collaboration with others and was also confirmed in that participants considered it important and appreciated the possibility to chat with physicians and nurses. In addition, they asked for opportunities to contact other patients and to be able to not only chat with health care personnel but also to book a time to see their physicians. Text-based communication with health care professionals also raised some concerns for those who were unfamiliar with it—what would be an understandable level? In addition, the difference between synchronous and asynchronous communication with physicians or nurses was not clear to all participants.

In addition, as is common to each health information technology application, there are issues associated with security and privacy, which we introduced as a fifth principle because of the usability testing. Patient portals, as clinical information systems, are complex environments that integrate patients’ data, information technologies, and human stakeholders. In designing patient portals optimally, it is imperative to protect patient confidentiality and follow health care provider guidelines [45]. Researchers proposed that the privacy principle should be classified into different levels according to importance [46]. To date, in Iran, there is no legal framework in use when it comes to information privacy, but there are legal provisions for cybercrime and protection of personal data [47]. The need to study the European General Data Protection Regulation is also felt in the Iranian legal system. Privacy and individuality are important ethical values and should be respected and protected in the design process [48]. In addition, privacy is gaining more attention in the design of communication technologies [49,50]. Finally, we would like to summarize the elements that support patient engagement. Helpful educational resources, supportive tools for planning and reminders, autonomy in task completion, and easy access to provider communication all encourage and sustain patient portal use for self-management.

### Comparison With Prior Work

As designers, there is a need to balance the strengths and weaknesses of a technology [23]. Some of these issues are recognized from previous studies, such as users asking for materials in everyday language without medical jargon while simultaneously requesting accurate and complete information about medical conditions and treatments [28,51]. Such contradictory requirements demand that designers consider the trade-offs. Another such trade-off is, on the one hand, building a web-based educational portal with information about the patient that is as complete as possible, and, on the other hand, burdening patients with having to enter data continuously and also having to worry about the integrity of their personal data, also shown in a study by Derboven et al [24]. Furthermore, the study identified how patients’ digital and health literacy and a perception of empowerment were crucial for them to be able to take responsibility for their self-management, which is in line with other studies [14,26-28]. Technology can help patients with chronic diseases take control of their conditions, but research needs to ensure that the technology’s design is perceived as safe [23]. In addition, research has previously identified technology as a third agent when generating personalized advice for self-management, which is something that can be automated [24].

This study shows that although design research is time-consuming, usability testing and severity ratings provide invaluable information for design. Going through the scenario and tasks made it possible for participants to indicate which features of the web-based educational portal did not work as intended. It is natural for a usability test to discover problems and glitches—users comment on difficulties rather than when the design works smoothly. A well-known fact in usability design is that users comment more on faults than on features that work well, as the former are unexpected, disturb interactions, and are easier to detect [52]. However, participants also suggested how to improve the design of the portal to better fit their needs. In addition, many of these usability problems can, in most cases, be fixed with a simple redesign, as is reported elsewhere [53]. Inappropriate cultural assumptions and low-level usability issues may, in many cases, lead to nothing more than just annoyance, but seemingly trivial design flaws may ultimately also lead to patient harm [54]. Most of the usability problems that participants encountered in this study concerned entering the data in tasks 1 and 2, which were also found by Mazaheri Habibi et al [55]. Entering personal information and health data on this kind of portal may be a daunting task for patients, and they may also feel insecure about filling in the right data. Developing guidelines to help patients with registration is therefore a necessity [56]. It remains unclear, especially considering the varying levels of digital and health literacy, whether such guidelines should be provided online, in printed form, or face-to-face. All the participants in this study successfully completed all the tasks, but most of the participants needed help along the way. Similar results were also found in studies of other patient groups [57]. Navigating a health portal is not a straightforward task, and efforts may be needed to produce clearer guidelines in order for portals to be used in practice. A section explaining any changes and improvements regarding the various functionalities can be helpful [15]. One particular difficulty in this case was that while the portal was in Farsi, participants were required to enter their laboratory results in English. Previous studies have shown that patients may find medical terminology on portals difficult [58-60], and they may need more guidance for such interactions. Research has also shown that participants with limited health literacy experience more barriers to using patient portals [56].

Our findings appear to be consistent with prior evaluations of diabetes self-management technologies in similar contexts. Nabovati et al [61] reported high usability for a diabetes app that combined educational content with calculators, reminders, graphical feedback, and physician communication. Our participants similarly endorsed those functions as motivating and practically helpful. Salari et al [62] further showed that personalization guided by behavioral theory and a clinician portal can promote independence and keep users involved. Complementing these results, Ghodousi Moghadam et al [63] demonstrated that a mobile serious game can enhance motivation for diabetes self-management. Together, these studies reinforce our conclusions that educational resources, planning or reminder tools, autonomous task support, and provider communication facilitate sustained portal use, while also reflecting the barriers we found, that is, the need to avoid complex language and to remediate confusing interactions through iterative, user-centered design.

One core aspect of usability is how effectively a design supports users in reaching their desired goal, in this case, self-management. This think-aloud study has investigated aspects that either support or risk being barriers to patients’ self-management. Many usability problems and challenges regarding self-management were identified, including negative examples that seem to contradict the design principles. Such observations, however, enable a better understanding of the design principles. The findings enable the principles to be refined to better reach their goals, and the observations from the study, in combination with the input from the participants, have provided several possible ways of addressing these issues.

### Limitations

Only literature incorporated in the authors’ previous research informed the development of the framework of design principles. Consequently, the range of identified principles may have been influenced by this limited evidence base. Then, this study focused on a group of patients with T2D at a diabetic clinic in one specific city, Mashhad, which may not fully capture the diversity of patients in other settings or geographical locations. This limitation could affect the generalizability of the results. The study also concentrated on specific tasks within the portal’s 9 sections, potentially overlooking usability issues or interactions between different sections. To address these gaps, future research should involve more clinical settings and a wider sample of patients. Nevertheless, the research was helpful in refining a framework of design principles pertaining to the design of web-based resources for the self-management of patients with T2D.

### Conclusions

A portal designed to enhance patients’ understanding, motivation, autonomy, and interaction with health care personnel is likely to be highly appreciated and to effectively support self-management. However, the design must also rigorously address risks that can undermine adoption and sustained use. Poorly designed interactions, such as confusing navigation, unclear workflows, or unintuitive interfaces, can hinder patients’ ability to use the portal effectively. Likewise, complex or technical language may reduce comprehension, discourage use, and limit the portal’s value for individuals with varying levels of health literacy. In addition, robust safeguards are required to protect patient integrity and data security, as trust in the system is essential for meaningful engagement.

We have proposed a framework of design principles pertaining to the design of web-based resources for the self-management of patients with T2D. Our results imply that usability testing, despite being time-consuming, is necessary when assessing a web-based educational portal, its potential for patients, and its sustained long-term use.

## Supplementary material

10.2196/78903 Multimedia Appendix 1 Reporting checklist for our study.

10.2196/78903 Multimedia Appendix 2 Instructions to participants.

10.2196/78903 Multimedia Appendix 3 Ethical approval.

## Abbreviations

QoL: quality of life
T2D: type 2 diabetes
