# Supplementary material for: Aspects Supporting and Hindering Type 2 Diabetes Self-Management in Web-Based Educational Portals: Usability Testing Study With Updated Framework in Razavi-Khorasan, Iran
Source: JMIR Hum Factors. 2026 Apr 1;13:e78903. doi: 10.2196/78903 (PMC13043003; doi:10.2196/78903)
Supplement: Multimedia Appendix 1 [file humanfactors-v13-e78903-s001.pdf]

**Checklist of procedures for planning and reporting procedures for usability evaluation with users**

| Subject | Item no | Checklist item | Reported on page No |
|---|---|---|---|
| Usability assessment moderator | 1a | "Specify as inclusion criteria having previous experience with usability evaluation with users or consider adequate training and provide details of the training plan." [1] | 3 |
| | 1b | "Specify whether the usability assessment moderator(s) is external to the service or product development team." [1] | 3 |
| | 1c | "Specify if observers are included, define their responsibilities, and collect their characteristics (e.g., gender, academic background, and previous experience in usability evaluation)." [1] | 3 |
| Participants | 2a | "Determine sample size (i.e., the total number of participants involved in the evaluation)." [1] | 3 |
| | 2b | "Provide a rationale to establish the sample size." [1] | 3 |
| | 2c | "Provide clear inclusion and exclusion criteria (e.g., profile definition including age, gender, educational level, digital literacy, previous experience using the product or service being evaluated)." [1] | 3 |
| | 2d | "Provide sampling methods (e.g., random, systematic, cluster, convenience, snowball)." [1] | 3 |
| | 2e | "Indicate the setting of participants' recruitment (e.g., community, hospital)." [1] | 3 |
| | 2f | "Detail clinical conditions (if relevant for the study): (e.g., asymptomatic or with a specific clinical condition or from a specific group - occupational group, the severity of the clinical condition, disabilities, cognitive impairment)." [1] | 3 |
| | 2g | "Detail the participant's characteristics that should be collected (such as age, gender, educational level, digital literacy)." [1] | 3 |
| Usability evaluation method and usability evaluation technique | 3a | "Specify whether a combination of usability evaluation methods is used (e.g., using both inquiry and test methods)." [1] | 3 |
| | 3b | "Specify whether a combination of usability evaluation techniques is used (e.g., for the inquiry method combine the questionnaire and interview techniques)." [1] | 3 |
| | 3c | "Provide the rationale for the choice of usability evaluation method(s) and technique(s)." [1] | 3 |
| | 3d | "Describe the usability evaluation method(s) and technique(s) used and how they are implemented." [1] | 3 |

| | | | |
|---|---|---|---|
| | 3e | "When using measuring instruments such as scales or questionnaires, give indicators of their validity and reliability." [1] | 3 |
| | 3f | "Describe the data analysis plan for both quantitative and qualitative data." [1] | 5 |
| Tasks | 4a | "Provide a detailed description of tasks or present the session script." [1] | 4,5 |
| | 4b | "Indicate the total number of tasks." [1] | 4 |
| | 4c | "Detail the tasks related outcomes and how they are measured (e.g., task completion and duration, and a number of errors)." [1] | Table 2, Figure 2 |
| | 4d | "Detail the conditions for carrying out the tasks (e.g., with or without supervision, individually or in a group, with or without a period for familiarization with the digital product or service)." [1] | 4 |
| | 4e | "Detail the instructions to participants and the way they are presented (e.g., verbally; written, both) and registered (e.g., audio, video, screen recorder, notes from an observer)." [1] | Multimedia Appendix 2, 4 |
| Usability evaluation environment | 5a | "Justify the choice of the usability evaluation environment (e.g., lab or field test; remote or face-to-face test)." [1] | 5 |
| | 5b | "Specify usability evaluation environment requirements (e.g., recording equipment or observer room availability)." [1] | 5 |

**Reference**


1. Supplementary material of: Martins AI, Santinha G, Almeida AM, Ribeiro Ó, Silva T, Rocha N, et al. Consensus on the Terms and Procedures for Planning and Reporting a Usability Evaluation of Health-Related Digital Solutions: Delphi Study and a Resulting Checklist. J Med Internet Res. 2023;25:e44326. doi:10.2196/44326. PMID: 37279047.
