# Supplementary material for: Aspects Supporting and Hindering Type 2 Diabetes Self-Management in Web-Based Educational Portals: Usability Testing Study With Updated Framework in Razavi-Khorasan, Iran
Source: JMIR Hum Factors. 2026 Apr 1;13:e78903. doi: 10.2196/78903 (PMC13043003; doi:10.2196/78903)
Supplement: Multimedia Appendix 2 [file humanfactors-v13-e78903-s002.pdf]

Scenario - Instructions to participants

1. The user registers in the Self-care.ir system, receives a login code, and enters their personal information into the portal using that code.
2. The user records their existing laboratory test results in the system.
3. The user accesses the training courses, completes the initial pre-test questions for each topic, and reviews the corresponding educational materials.
4. The user reads educational content related to diabetes and its effects on different body organs.
5. Using the “Help me” section, the user proceeds step-by-step through guided instructions to address a specific problem or concern.
6. The user views their registered test data in the Health Progress section, including visual charts of trends over time.
7. The user opens external links and watches educational videos provided within the portal.
8. The user calculates their ideal weight and daily calorie requirements and selects appropriate food items from the diet list.
9. The user engages in online communication with a doctor or nurse through the portal’s correspondence feature.
