# Supplementary material for: Aspects Supporting and Hindering Type 2 Diabetes Self-Management in Web-Based Educational Portals: Usability Testing Study With Updated Framework in Razavi-Khorasan, Iran
Source: JMIR Hum Factors. 2026 Apr 1;13:e78903. doi: 10.2196/78903 (PMC13043003; doi:10.2196/78903)
Supplement: Multimedia Appendix 3 [file humanfactors-v13-e78903-s003.pdf]

Ref : 306653 :شماره

Date : .... : تاریخ

Mashhad University of Medical Sciences

دانشگاه علوم پزشکی مشهد

معاونت پژوهشی

Vice Chancellor for Research

28th May 2016

Reference Number: IR.MUMS.REC.1395.108

Mashhad-Iran
P.O. Box 951

To Whom It May Concern:

This is to certify that the research protocol entitled:
"Exploring the process of web-based education in diabetic patients: Designing educational model "
Has been submitted to the Ethical Committee of Mashhad University of Medical Sciences. Upon reviewing, the Ethical Committee approved the above mentioned protocol in its session hold on "21th May 2016". The Committee did not encounter any deviation from ethical principals in the methodological aspect of the research. 4716280

Yours Sincerely,

Mohsen Tafaghodi, PhD

Vice-Chancellor for Research

MASHHAD-IRAN
Tel : +98 511 8411538
P.O.Box :91735-951
Fax:+98 511 8430249
www.mums.ac.ir/research
E-mail:vcresearch@mums.ac.ir
